# High prevalence, intensity, and genetic diversity of *Trichinella* spp. in wolverine (*Gulo gulo*) from Yukon, Canada

**DOI:** 10.1186/s13071-021-04636-2

**Published:** 2021-03-08

**Authors:** Rajnish Sharma, N. Jane Harms, Piia M. Kukka, Thomas S. Jung, Sarah E. Parker, Sasha Ross, Peter Thompson, Benjamin Rosenthal, Eric P. Hoberg, Emily J. Jenkins

**Affiliations:** 1 Department of Veterinary Microbiology, Western College of Veterinary Medicine, University of Saskatchewan, 52 Campus Drive, Saskatoon, SK S7N 5B4 Canada; 2 Department of Environment, Government of Yukon, P.O. Box 2703, Whitehorse, YT Y1A 2C6 Canada; 3 Department of Renewable Resources, University of Alberta, 351 General Services Building, Edmonton, AB T6H 3T1 Canada; 4 Centre for Applied Epidemiology, Large Animal Clinical Sciences, Western College of Veterinary Medicine, University of Saskatchewan, 52 Campus Drive, Saskatoon, SK S7N 5B4 Canada; 5 USDA-Agricultural Research Service, Animal Parasitic Diseases Laboratory, Beltsville Agricultural Research Center, 10300 Baltimore Avenue, Beltsville, MD 20705 USA; 6 Museum of Southwestern Biology and Department of Biology, University of New Mexico, Albuquerque, NM 87131-0001 USA; 7 Department of Pathobiological Sciences, School of Veterinary Medicine, University of Wisconsin-Madison, Madison, WI 53706 USA

**Keywords:** *Trichinella* spp., Wolverine, Canada, Prevalence, *T. nativa*, *Trichinella* T6, *T. chanchalensis*

## Abstract

**Background:**

Species of *Trichinella* are globally important foodborne parasites infecting a number of domestic and wild vertebrates, including humans. Free-ranging carnivores can act as sentinel species for detection of *Trichinella* spp. Knowledge of the epidemiology of these parasites may help prevent *Trichinella* spp. infections in northern Canadian animals and people. Previous research on *Trichinella* spp. in wildlife from Yukon did not identify risk factors associated with infection, or the diversity and identity of species of *Trichinella* in regional circulation, based on geographically extensive sampling with large sample sizes.

**Methods:**

In a cross-sectional study, we determined the prevalence, infection intensity, risk factors, and species or genotypes of *Trichinella* in wolverine (*Gulo gulo*) in two regions of Yukon, Canada, from 2013–2017. A double separatory funnel digestion method followed by mutiplex PCR and PCR-RFLP were used to recover and identify species of *Trichinella*, respectively.

**Results:**

We found larvae of *Trichinella* in the tongues of 78% (95% CI 73–82) of 338 wolverine sampled. The odds of adult (≥ 2 years) and yearling (1 year) wolverine being *Trichinella* spp.-positive were four and two times higher, respectively, compared to juveniles (<1 year). The odds of *Trichinella* spp*.* presence were three times higher in wolverine from southeast than northwest Yukon. The mean intensity of infection was 22.6 ± 39 (SD, range 0.1–295) larvae per gram. *Trichinella* T6 was the predominant genotype (76%), followed by *T. nativa* (8%); mixed infections with *Trichinella* T6 and *T. nativa* (12%) were observed. In addition, *T. spiralis* was detected in one wolverine. Out of 22 isolates initially identified as *T. nativa* in multiplex PCR, 14 were analyzed by PCR-RFLP to distinguish them from *T. chanchalensis*, a recently discovered cryptic species, which cannot be distinguished from the *T. nativa* on multiplex PCR. Ten isolates were identified either as *T. chanchalensis* alone (*n* = 7), or mixed infection with *T. chanchalensis* and *T. nativa* (*n* = 2) or *T. chanchalensis* and *Trichinella* T6 (*n* = 1)].

**Conclusions:**

Wolverine hosted high prevalence, high larval intensity, and multiple species of *Trichinella*, likely due to their scavenging habits, apex position in the food chain, and wide home range. Wolverine (especially adult males) should be considered as a sentinel species for surveys for *Trichinella* spp. across their distributional range.
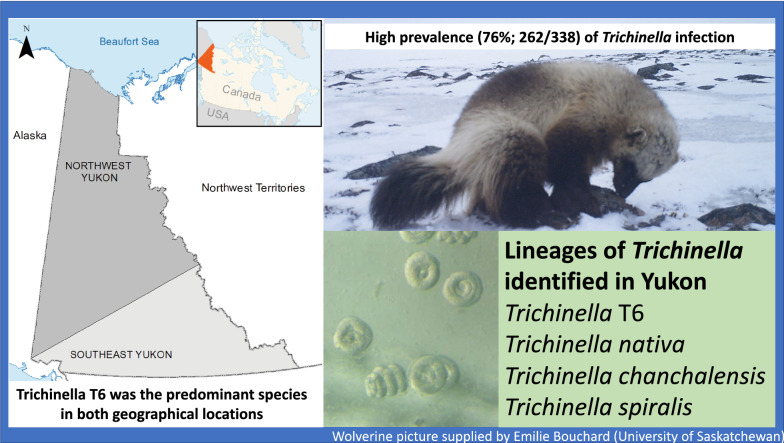

## Background

Species of *Trichinella* nematodes are among the most significant foodborne parasites listed by the World Health Organization/United Nations Food and Agriculture Organization [1]. All *Trichinella* species and genotypes have been reported in mammals, whereas *T. pseudospiralis* also develops in avian hosts, and *T. papuae* and *T. zimbabwensis* have also been reported from reptilian hosts. All species and genotypes of *Trichinella* are zoonotic and transmitted by consuming raw or insufficiently cooked meat [2]. There are 13 species of *Trichinella* classified into two clades: encapsulated and nonencapsulated. A survey in Canada conducted over a period of 10 years on 15 wildlife species showed an overall prevalence of 20% for multiple species of *Trichinella* among tested animals [3], and identified *T. nativa, Trichinella* T6, *T. murrelli*, and *T. pseudospiralis* [3]*. Trichinella chanchalensis,* a cryptic encapsulated species, has recently been discovered and described in wolverine (*Gulo gulo*) from northwestern Canada [4].

Globally, most human outbreaks of trichinellosis have been attributed to domestic pigs infected with *T. spiralis* [5]*.* However, almost all human outbreaks in the last 20 years in Canada involved *T. nativa* and *Trichinella* T6 linked to consumption of raw or insufficiently cooked game meat, commonly from walrus (*Odobenus rosmarus*) and bears (*Ursus* spp.) [6–9]. For example, 15 trichinellosis outbreaks have occurred in Nunavik (northern Québec, Canada) from 1982–2009; of these, nine were linked to walrus meat [9]. One of the largest outbreaks reported in Canada was from Saskatchewan, where 78 people became sick after consuming meat from a black bear (*Ursus americanus*) infected with *T. nativa* [7]. A recent outbreak of human trichinellosis due to *T. nativa* in Ontario, Canada was associated with consumption of dried meat of black bear and resulted in rare thrombotic complications [8]. *Trichinella* T6 has also been identified in an outbreak in Ohio, USA, linked to consumption of bear meat from Canada [6]. Knowledge of the epidemiology of the disease and pathways for exposure from wild animals can help prevent *Trichinella* spp. infections in humans and animals.

Free-ranging carnivores may be reservoirs of *Trichinella* spp., and may act as sources of infection for humans and as sentinel species for parasite detection. For example, various species of canids have been previously proposed as sentinel species for *Trichinella* spp. [10–12]. An ideal sentinel animal host should possess the following features: adequate availability (relative population stability), measurable response (such as parasites in tissues), earlier response than people or sympatric wild species (high chance of exposure early in life and a relatively short life span), and high levels of exposure [13, 14]. Wolverine (*Gulo gulo*) may act as an ideal sentinel species for detecting *Trichinella* spp. in northwestern North America because (1) they are legally killed for their fur and carcasses are available from fur harvesters [15], (2) initial studies have reported relatively high prevalence and intensity of *Trichinella* spp. infection in wolverine, suggesting that they may be a key species in the epidemiology of infection [3, 16], and (3) they are a facultative scavenger [17–19] near the top of terrestrial food webs, and as such may be bio-accumulators of foodborne parasites such as *Trichinella* spp.

There are few published studies on species of *Trichinella* infecting wild carnivores from the Yukon [3, 20, 21]. The two older studies (45 years ago) reported *Trichinella* spp. in 71% of 21 grizzly bear (*Ursus arctos*) from the Yukon [21] and 47% of 127 gray wolves (*Canis lupus*) from Yukon and the Northwest Territories [20]. Larvae of *Trichinella* spp. were detected in Arctic fox (*Vulpes lagopus*), wolf, and wolverine from the Yukon in a survey conducted 10 years ago [3]. Previously, we published two studies, one where we compared tongue and diaphragm muscles as predilection sites [22] and another where we discovered a new species of *Trichinella* [4] in a portion of wolverine carcasses used in the present study. Previous research on *Trichinella* in wildlife from Yukon did not involve geographically extensive and site-intensive sampling, or large sample sizes. To date, no epidemiological study has associated risk factors or identified recovered larvae to species level. To fill these knowledge gaps, we sampled a large number of wolverine across Yukon, Canada.

To determine the suitability of wolverine as a sentinel species, we therefore sought (1) to identify the species/genotypes of *Trichinella* and their distribution in Yukon; (2) to determine the prevalence and intensity of infections with species of *Trichinella*; and (3) to explore the associations between potential risk factors and *Trichinella* spp*.* positivity and larval intensity in wolverine.

## Methods

### Wolverine sampling

**Fig. 1:**
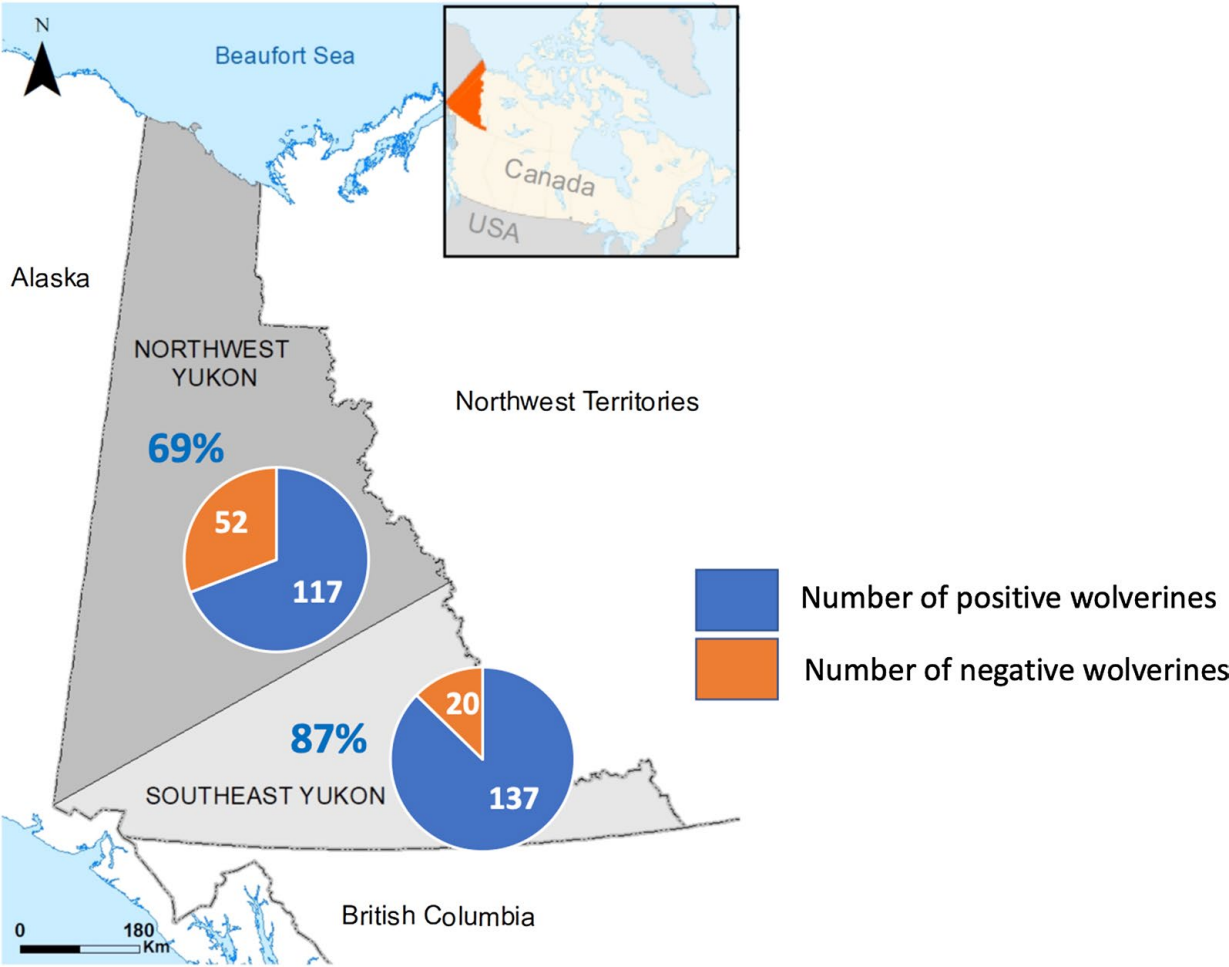
Broad phylogeographic regions (northwest and southeast) of wolverine (*Gulo gulo*) populations in Yukon, Canada

We obtained wolverine carcasses from across Yukon, Canada, which is ~ 483,000 km^2^ and sparsely populated by people (~ 0.08 people\km^2^), with 76% of the human population living in the city of Whitehorse [23]. The topography of Yukon is rugged and characterized by mountain ranges, plateaus, valleys, and lowlands. Climate is subarctic continental, with annual precipitation ranging from 250–600 mm, most of which falls as snow from October to May. The mean daily temperature ranges from −15 °C to −30 °C in January and from 10 °C to 15 °C in July. Yukon is characterized by boreal forest in valley bottoms, and shrub communities and alpine tundra at and above the treeline, respectively [24]. Wolverine are legally harvested throughout Yukon by fur trappers during the winter [25–27], with an average of 132 ± 31 (standard deviation [SD]) animals harvested annually [25]. Wolverine carcasses were submitted by fur trappers to Environment Yukon (Government of Yukon) and kept frozen at −20°C for 6–10 months prior to necropsy. The harvest location and sex of each animal were recorded, and age was determined by cementum analysis of a premolar tooth at a commercial laboratory (Matson’s Laboratory LLC, Milltown, MT, USA). Wolverine were classified as juveniles (< 1 year), yearlings (1 year), or adults (≥ 2 years) based on cementum-derived ages. A body condition index (BCI) for each wolverine was calculated using predetermined regression equations for wolverine from our study area that incorporated the mass of select fat depots with body size and sex, where higher BCIs corresponded to greater mass-specific fat levels, for each sex, than lower BCIs [27]. Tongues were collected in plastic bags and stored at −20 °C until further analyses. The harvest location of each wolverine was assigned to one of two broad geographic areas (northwest Yukon or southeast Yukon; Fig 1), because these geographic areas correspond to broad phylogeographic divisions for wolverine that could influence the genetic diversity of wolverine parasites [28, 29].

The number of wolverine in our study area was unknown but was estimated to range between 3500 and 4000 [30]. We used OpenEpi version 3.01 [31] to estimate the sample size necessary to be representative of the wolverine population in Yukon. We parameterized OpenEpi by using the midpoint of the population size estimate range (3750) and an expected prevalence of *Trichinella* spp. of 80%, based on earlier reports of 88% and 77% [3, 16] in wolverine from northern Canada. Based on these values, the calculated sample size necessary for our study was 231 with a confidence interval of 95%. We obtained 338 wolverine carcasses from animals harvested during 2013–2017, which exceeded the estimated sufficient sample size for our study.

Because we obtained samples from animals that were harvested for purposes other than our research, this was considered exempt from animal care committee review at the University of Saskatchewan. We obtained a wildlife research permit and appropriate export permits from the Government of Yukon.

### Recovery of *Trichinella* spp. larvae

In a previous study, we compared tongues and diaphragms obtained from wolverine harvested in 2012–2013 and 2013–2014  and found that the tongue was a better predilection site for *Trichinella* spp. [22]. Thus, only tongues were collected for recovery of larvae in the current study. Tongues were thawed at room temperature and cut into 0.5–1.0 cm cubes, which were mixed and a portion randomly selected to make up to 10 g. Connective tissue and fat were removed and ≤10 g of muscle was digested using the double separatory funnel digestion method [32]. Larvae per gram (LPG) of each positive sample were calculated by dividing total number of larvae by weight of digested tissue. To identify *Trichinella* species, larvae were collected in 1X polymerase chain reaction (PCR) buffer and stored at −20 °C until DNA extraction [33].

### Molecular identification of *Trichinella* spp. by multiplex PCR

From each wolverine positive for *Trichinella* spp., parasite genomic DNA was extracted from five individual larvae as well as a pool of ten larvae using a Proteinase K extraction method [33, 34]. In the case of availability of less than 15 larvae from a positive animal, five individual larvae and the rest of the larvae in the pool were processed. To identify species or genotypes of *Trichinella* larvae, primers amplifying internal transcribed spacer regions (ITS 1 and 2) as well as the expansion segment V of the large subunit ribosomal DNA were used in a multiplex PCR assay, as previously described [33, 34]. PCR reactions were performed with master mix, containing 1X AmpliTaq Gold 360 Master Mix (Applied Biosystems, CA, USA), 50 μM primers, 2 μl of GC Enhancer, 2.5 μl of DNA, and ultrapure water to a total volume of 25 μl. The thermocycler (Bio-Rad, USA) conditions were 95 °C for 10 min followed by 40 cycles of 95 °C for 15 s, 55 °C for 30 s, 72 °C for 90 s, and final elongation at 72 °C for 7 min. Species were identified based on the band pattern developed on 2.5% agarose gel when analyzed under UV light using a gel doc system (Alpha Innotech AlphaImager digital imaging system). DNA from six species of *Trichinella* (*T. spiralis*, *T. nativa*, *T. britovi*, *T pseudospiralis*, *T murrelli*, and *Trichinella* T6) were used as positive controls. Positive controls, negative template control, and DNA extraction control (only 10X PCR buffer) were also included in each PCR. *Trichinella nativa* and *T. chanchalensis* showed similar banding patterns on the multiplex PCR (a band of 127 bp); therefore, a subsequent PCR-restriction fragment length polymorphism (RFLP) was performed to differentiate them, as previously described [4].

### Data analysis

A wolverine was considered positive for *Trichinella* spp. if ≥1 larva was recovered from tissue samples. Using Epitools epidemiological calculators [35], prevalence with 95% confidence intervals (CI) was calculated from the proportion of wolverine that were positive for any species of *Trichinella*. The intensity of infection was measured as LPG, with “low” and “high” intensity defined as ≤10 LPG and >10 LPG, respectively. We built binary logistic regression models to independently test for (1) presence of *Trichinella* spp. (present or absent) and (2) intensity of *Trichinella* spp. infection (low or high). We evaluated the following potential predictors for inclusion in a final multivariable model using univariable logistic regression analysis with a relaxed level of significance (*p* ≤ 0.20): age (juvenile, yearling, or adult), sex (female or male), harvest location (southeast Yukon or northwest Yukon), harvest season (winters 2013–2014, 2014–2015, 2015–2016, or 2016–2017), and BCI (continuous variable). The weight of muscle tissue processed (< 5 g, 5.0–9.9 g, or ≥10 g) was also included as a potential predictor in both models. For outcome “*Trichinella* spp. larval intensity (high/low),” single vs. co-infection status was also included as a predictor (single infection means infection with any one of the species *Trichinella* T6, *T. nativa*, *T. chanchalensis*, or *T spiralis*; co-infection means co-infection with either *Trichinella* T6 and *T. nativa, Trichinella* T6 and *T. chanchalensis*, or *T. nativa* and *T. chanchalensis*). Stepwise forward multivariable logistic regressions were performed to build the final model. Goodness of fit of the final model was evaluated by the Hosmer–Lemeshow test. Variables with a significance level of *p* < 0.05 were retained in the model. To estimate the degree of the association between each predictor and *Trichinella* status, odds ratios and respective 95% confidence intervals were calculated. All statistical analyses were performed using IBM SPSS software (version 24; Armonk, NY, USA).

## Results

### Descriptive analysis

The mean age and BCI of sampled wolverine was 1.6 years (SD = 2.05, range < 1–10) and 8.88 (SD = 5.24, range < 1–30), respectively. Age was not estimated for 3 of 338 wolverine (0.88%), and harvest location was not available for 12 of 338 wolverine (3.5%). Most carcasses submitted were male, with a male-to-female ratio of 2:1. The age-class distribution of wolverine that we sampled was similar among juveniles (37%), yearlings (29%), and adults (34%). Wolverine were obtained in relatively equal percentages (23–27%) from each of the 4 years of sampling as well as from the two phylogeographic regions; 50% and 46% from the northwest and southeast Yukon, respectively.

### *Trichinella* spp. prevalence

*Trichinella* spp. larvae were detected in 78% (262 of 338) of wolverine tested (95% CI 73–82). Larvae of *Trichinella* spp. were detected most frequently in adult wolverine (87%, 99 of 114, 95% CI 79–92), followed by yearlings (82%, 79 of 96, 95% CI 73–89) and juveniles (65%, 81 of 125, 95% CI 56–73). More males (80%, 95% CI 74–84) than females (73%, 95% CI 64–81) were positive for *Trichinella* spp. (Table 1).

**Table 1:** Prevalence (% positive) of *Trichinella* spp. in 338 wolverine (*Gulo gulo*) in Yukon, Canada

	Number of positive wolverine	Total animals examined	Prevalence	95% confidence interval
Age				
Juvenile	81	125	64.8	56.1–72.6
Yearling	79	96	82.3	73.5–88.6
Adult	99	114	86.8	79.4–91.9
Sex				
Female	82	112	73.2	64.3–80.6
Male	180	226	79.6	73.9–84.4
Harvest location				
Northwest	117	169	69.2	61.9–75.7
Southeast	137	157	87.3	81.1–91.6
Harvest year				
2013–2014	69	86	80.0	70.6–87.3
2014–2015	66	91	72.5	62.6–80.6
2015–2016	60	77	77.9	67.5–85.7
2016–2017	67	84	79.8	70.0–87.0
Weight of tongue processed				
Less than 5 g	24	36	66.7	50.3–80.0
5–9.9 g	22	28	78.6	60.5–89.8
10 g	216	274	78.8	73.6–83.3

Our univariable logistic regressions revealed that age (*p* < 0.001), sex (*p* = 0.184), and phylogeographic region (*p* < 0.001) were significantly associated with the presence of *Trichinella* spp. in wolverine. Our stepwise multivariable regression suggested that age and phylogeographic region were significantly associated with *Trichinella* spp. prevalence (Table 2). The odds of *Trichinella* spp. presence were two times (odds ratio = 2.25, 95% CI 1.16–4.36; *p* = 0.016) and four times (odds ratio = 3.76, 95% CI 1.86–7.60; *p* = 0.001) higher in yearlings and adults, respectively, than in juveniles (< 1 year). The odds of the presence of *Trichinella* spp. were three times (odds ratio = 2.87, 95% CI 1.60–5.16; *p* = 0.001) higher in wolverine from southeast Yukon than northwest Yukon.

**Table 2:** Risk factors for *Trichinella* spp. prevalence and intensity in wolverine (*Gulo gulo*) identified in final stepwise logistic regression; odd ratios (OR) and its 95% confidence intervals and probability (*p*)

Model (outcome) (*n*)	Predictors in the final model		OR	95 CI	*p*
*Trichinella* spp. infection (yes/no) [323]	Age	Juvenile			0.001
		Yearling	2.25	1.16–4.36	0.016
		Adult	3.76	1.86–7.60	0.001
	Harvest location	Northwest			0.001
		Southeast	2.87	1.60–5.16	0.001
*Trichinella* spp. larval burden (high/low) [254]^a^	Age	Adult			0.012
		Yearling	2.41	1.28–4.54	0.007
		Juvenile	1.07	0.57–2.00	0.840
	Single vs. co-infection status^b^	*Trichinella*-single species infection			0.002
		*Trichinella*-mixed infection	3.33	1.53–7.24	

*n* Animals with complete record for respective model

^a^Only positive samples with *Trichinella* species were included in the model

^b^Single infection means infection with any one of the listed species *Trichinella* T6, *T. nativa*, *T. chanchalensis*, or *T spiralis*; co-infection means co-infection with either *Trichinella* T6 and *T. nativa*, *Trichinella* T6 and *T. chanchalensis*, or *T. nativa* and *T. chanchalensis*

### Intensity of *Trichinella* spp. infection

The median intensity of *Trichinella* spp. larvae was 8.4 (range 0.1–295) LPG. Larval intensity > 1 was observed in 89% (232 of 262 animals sampled) wolverine. The median larval intensity of *Trichinella* spp. was higher in juveniles (14 LPG) than in yearlings (7 LPG) and adult wolverine (7 LPG). Age and co-infection was significantly associated with high larval intensity in both univariable and stepwise multivariable regression (Table 2). The odds of high larval intensity were two times (odds ratio = 2.41, 95% CI 1.28–4.54; *p* = 0.012) higher in yearlings than adult wolverine. The odds of high larval intensity were three times (odds ratio = 3.33, 95% CI 1.53–7.24; *p* = 0.002) higher in wolverine that were co-infected than those infected with a single species of *Trichinella* (Table 2).

### Genetic diversity of *Trichinella* spp.

Using multiplex PCR, *Trichinella* spp. larvae from 254 of 262 (97%) infected wolverine were identified to species/genotype. Overall, 85% (222 of 262) of wolverine were infected with a single species (*Trichinella* T6, *T. nativa*, or *T. spiralis*). *Trichinella* T6 was the predominant genotype (76%; 199 of 262), followed by *T. nativa* (8%; 22 of 262). Mixed infections with both *Trichinella* T6 and *T. nativa* were detected in 12% (32 of 262). Larvae of *T. spiralis* were present in one wolverine. Out of 22 pure *T. nativa* positive isolates (without any indication of co-infection on multiplex PCR), 14 were subjected to RFLP (DNA was not amplified from 8 isolates due to either disintegrated larvae or lack of sufficient larvae). Among the samples tested by RFLP, ten were positive for *T. chanchalensis*, with only three having mixed infection with either *T. nativa* or genotype T6. (Table 3). Overall (based on multiplex PCR and/PCR-RFLP), 86% (219/254) of wolverine were infected with a single species (either *Trichinella* T6 or *T. nativa* or *T. chanchalensis* or *T. spiralis*) and 14% were co-infected with two species (either T2 + T6, T2 + T13, T6 + 13).

**Table 3:** Species of *Trichinella* identified in wolverine (*Gulo gulo*) in the Yukon

Species diagnosis	By multiplex PCR	By PCR-RFLP^a^
*Trichinella* T6 only	199	NA
*T. nativa* only	22	4 (*T. nativa* only)
		7 (*T. chanchalensis* only)
		2 (*T. chanchalensis* and *T. nativa*)
		1 (*T. chanchalensis* and *Trichinella* T6)
		8 NA
*Trichinella* T6 and *T. nativa*	32	NA
*T. spiralis*	1	NA
Species not identified	8	NA

*NA* isolates of *Trichinella* not tested by PCR-RFLP

^a^Only 14 of 22 *T. nativa* isolates (identified on multiplex PCR) were tested by PCR-RFLP [4]

## Discussion

We report high prevalence (78%) of *Trichinella* spp. (predominantly T6) in wolverine in Yukon, Canada, based on the largest sampling effort yet for this species (*n* = 338 wolverine). The prevalence of *Trichinella* spp. appears to be substantially higher in wolverine than all other carnivores tested so far in northern Canada or Alaska, with the possible exception of grizzly bears (*Ursus arctos*; Additional file 1: Table S1). The prevalence of *Trichinella* spp. in wolverine in our study was comparable to other reports from northern Canada, where prevalence of 77% and 88% had been noted [3, 16], and higher than that observed for this species in other countries (0% in Sweden, 30% in Kamchatka, Russia, 50% in Alaska, USA) [36–38] (Additional file 1: Table S1). Variation in prevalence documented across studies may be due to differences in sample size, type of muscle sampled (tongue, diaphragm, cheek muscles, or leg muscles), or methodology (trichinoscopy versus muscle digestion) [37, 39–41]. In the current study, we used the pepsin-HCl digestion method, which has higher sensitivity than trichinoscopy [37, 39, 40]. Digestion is now considered the gold standard for detection of larval *Trichinella* spp. in animal tissues [42]. We used tongue tissue, which has been demonstrated to be a more sensitive sampling site than the diaphragm for detection of larval *Trichinella* spp. in wolverine [22]. Finally, we used relatively large samples of tongue tissue for examination (89% of samples were ≥ 5 g), as recommended for studies of wild carnivores [43].

Our data indicate that prevalence increases with age, as demonstrated for other wildlife species in Europe [44, 45] but not for a similar study in wolverine from Nunavut, Canada [16]. The reason for higher prevalence of *Trichinella* spp. infection in older animals likely reflects an accumulation of diet-based exposure risk, and enduring chronic infection thereafter. A higher prevalence of *Trichinella* spp. in male than female wolverine was observed, consistent with findings from a similar survey in the Northwest Territories, Canada [46], whereas almost equal prevalence rates (89% in male vs. 86% in female wolverine) were reported in wolverine from Nunavut, Canada [16]. Males have larger home ranges, and disperse further, than females [47], increasing opportunities for exposure.

*Trichinella* spp. were widely distributed in wolverine in Yukon; however, prevalence was higher in the southeast region than in the northwest region. There may be regional variation in wolverine diet, which may play a role in varying prevalence among regions. Other factors that can affect regional differences in prevalence of *Trichinella* spp. may include environmental factors such as snow cover, altitude, air temperature or humidity, or anthropogenic factors. For example, the number of snow cover days was positively associated with the incidence of *Trichinella* spp. infections among wild boars from Latvia [48]. However, based on this assumption alone, a greater number of snow cover days in northern Yukon suggests that prevalence should have been higher in the northern regions.

In our study we observed a median infection intensity (8 LPG) that was twice as high as that reported from an earlier study in northwestern North America (including British Columbia, Northwest Territories, and Yukon; 3.7 LPG; Additional file 1: Table S2; [3]). In contrast to the differences in age-related prevalence, we observed higher median larval intensity in juveniles than yearlings and adults. This suggests that while risk of exposure increases with age, the severity of infection is higher in young animals, which could be due to juveniles having relatively undeveloped immunity.

We determined the genotype and/or species of *Trichinella* circulating among wildlife in Yukon. Our surveys revealed two sylvatic species of *Trichinella* (*Trichinella* T6 and *T. nativa*) and, unexpectedly, a species typically considered to be restricted to domestic circulation (*T. spiralis*). Moreover, *T. chanchalensis* (a previously unknown and cryptic species of *Trichinella*) was also reported, occurring at a greater prevalence in single infections than *T. nativa*, from which it must be distinguished by PCR-RFLP [4]*.*

Due to their freeze tolerance, larvae of *T. nativa*, *Trichinella* T6, and *T. chanchalensis* are expected in arctic and subarctic regions. We report higher prevalence of *Trichinella* T6 than *T. nativa* in wolverine from Yukon, consistent with observations elsewhere in the western Canadian Arctic (Nunavut) [16]. *Trichinella* T6 is the most common genotype of *Trichinella* observed in Canadian wildlife, with wider host and geographic ranges than *T. nativa* [3]. Differences in infectivity and host range among genotype or species of *Trichinella* warrant further study.

*Trichinella spiralis* is a reportable pathogen in domestic animals in Canada. Currently, Canada is considered free of *T. spiralis* in swine raised for commercial purposes, and a single case does not immediately implicate its circulation among free-ranging wildlife hosts. Although the source of infection remains unknown for this isolated observation, it is unlikely that wolverine play a significant role in transmission of *T. spiralis* (although see discussion about ecological fitting and otherwise incidental hosts in the circulation of parasites [49, 50]). Further, because *T. spiralis* is not freeze-tolerant, the significance of this finding remains uncertain. Additional studies are required to explore the degree to which wolverine and other wildlife may serve as reservoirs for *T. spiralis*.

Our results show that *Trichinella* T6, *T. chanchalensis*, and *T. nativa* occur in sympatry geographically (Yukon) and in the same individual host(s) (wolverine). Co-infection with *T. nativa* and *Trichinella* T6 has been previously reported in wolverine [16], and hybridization between the two species has been observed in Alaska [51]. The multiplex PCR (which targets nuclear DNA only) does not distinguish hybrids of *Trichinella* T6 and *T. nativa,* which requires microsatellite and/or mitochondrial DNA analysis [52]. Based on a study in wolverine from Nunavut, microsatellite and mitochondrial DNA analysis showed that hybrids appear as *T. nativa* on the multiplex PCR*.* We report mixed infection of *T. chanchalensis* with either *Trichinella* T6 or *T. nativa* as well.

Routes of transmission of *Trichinella* larvae among wildlife include scavenging, predation, and cannibalism [53–55]. The overall high prevalence, intensity, and diversity of *Trichinella* spp. in wolverinemay indicate that they become infected by multiple pathways. Hunting and fur trapping practices such as leaving carcasses in the field, or using meat of wild carnivores as bait, may facilitate transmission [56]. While clinical disease due to *Trichinella* is usually absent in animals, surveying wildlife is important in terms of zoonoses and food safety [50]. For example, well-organized testing programs in Nunavik (northern Québec, Canada) for *Trichinella* in walruses have been developed to limit the dissemination of infected meat before distribution among communities [9, 57]. Monitoring wildlife can also be important to determine epidemiological and genetic baselines for endemic *Trichinella* species, and to detect spillover of *T. spiralis* or introduced species into wildlife. For example, in Europe, especially in regions with no recognized *Trichinella* infection in pigs, monitoring of wildlife using suitable sentinel species is mandatory [58].

## Conclusion

Wolverine, especially adult males, are good sentinels for *Trichinella* spp. in northwestern North America because they have a high prevalence and diversity (at least 3 endemic species) of *Trichinella*. Moreover, carcasses obtained from fur trappers provide relatively large and consistent sample sizes, and engage local people in surveillance programs. As such, we suggest using wolverine for monitoring changes in *Trichinella* spp. infection in wildlife of northwestern Canada, and our data can be used as a baseline. Larval intensity ≥ 1 LPG is considered a significant risk for food safety [3], and 89% of wolverine positive for *Trichinella* spp. in our study met or exceeded this threshold. Although wolverine are not consumed as food by people, universal precautions (namely wearing gloves and thoroughly washing hands before eating, and storing wolverine carcasses away from other meat intended as food, and away from pets) should be practiced when handling wolverine carcasses to prevent transmission. Practices such as leaving carcasses in the field or using wolverine meat as bait for trapping or hunting may also facilitate transmission, even in winter, as larvae of sylvatic species of *Trichinella* present in arctic and subarctic regions survive freezing.

## Supplementary Information

**Additional file 1: Table S1.** Prevalence of *Trichinella* spp. in wild carnivores in Canada and Alaska. **Table S2.** Prevalence and larval burden (larvae per gram) of *Trichinella* spp. in wolverine from different geographical locations.

## Data Availability

The data sets generated and/or analyzed during the current study are available from the corresponding author on reasonable request.
