# Supplementary material for: High prevalence, intensity, and genetic diversity of Trichinella spp. in wolverine (Gulo gulo) from Yukon, Canada
Source: Parasit Vectors. 2021 Mar 8;14:146. doi: 10.1186/s13071-021-04636-2 (PMC7938582; doi:10.1186/s13071-021-04636-2)
Supplement: Supplementary file 1 — Additional file 1: Table S1. Prevalence of Trichinella spp. in wild carnivores in Canada and Alaska. Table S2. Prevalence and larval burden (larvae per gram) of Trichinella spp. in wolverine from different geographical locations. [file 13071_2021_4636_MOESM1_ESM.docx]

Table S1 Prevalence of *Trichinella* spp. in wild carnivores in Canada and Alaska

| **Host** | **Location** | **%** | **Total number tested** | **Genotype (Number of isolates of *Trichinella* spp. genotyped)** | **Reference** |
| --- | --- | --- | --- | --- | --- |
| Arctic fox (*Vulpes lagopus*) | NT | 3 | 1566 | ND | [1] |
|  | NU, NT | 11 | 28 | ND | [2] |
|  | AK | 10 | 117 | ND | [3] |
| Black bear (*Ursus americanus*) | NT | 6 | 120 | T2 (4), T6 (2) | [4] |
|  | QC | 1 | 107 | ND | [5] |
|  | BC, SK, QC, NT | 7 | 193 | BC- T2 (NM), SK-T2 (NM), QC-T2 (NM), NT- T2 (NM) &T6 (NM) | [2] |
|  | AK | 24 | 21 | ND | [3] |
|  | AK | 28 | 40 | ND | [6] |
| Bob cat (*Lynx rufus*) | NS | 4 | 24 | ND | [2] |
| Coyote (*Canis latrans*) | NS, BC | 1 | 346 | ND | [2] |
|  | AK | 13 | 8 | ND | [3] |
| Ermine (*Mustela erminea*) | AK | 43 | 40 | ND | [3] |
|  | AK | 9 | 11 | ND | [3] |
| Fisher (*Martes pennanti*) | BC, NS | 29 | 17 | ND | [2] |
| Grizzly Bear (*Ursus arctos*) | YT, NT | 88 | 24 | ND | [7] |
|  | NT | 73 | 11 | T6 (6), T2 & T6 (1) | [4] |
|  | BC, NU | 29 | 68 | T6 (NM) | [2] |
|  | AK | 50 | 20 | ND | [3] |
| Lynx (*Lynx canadensis*) | BC, NU | 7 | 107 | T6 (NM) | [2] |
|  | AK | 24 | 17 | ND | [3] |
| Marten (*Martes americana*) | BC, NU | 3 | 101 | ND | [2] |
| North American Cougar (*Puma concolor cougar*) | BC | 44 | 127 | BC (mainland) T2 (NM), T6 (NM), BC (Island) T4 (NM), T5 (NM) | [2] |
| Polar bear (*Ursus maritimus*) | QC, NU | 66 | 85 | T2 | [2] |
|  | AK | 53 | 17 | ND | [3] |
|  | AK | 47 | 478 | ND | [6] |
| Red Fox (*Vulpes vulpes*) | NT | 11 | 19 | ND | [1] |
|  | AB | 6 | 18 | ND | [1] |
|  | AK | 41 | 76 | ND | [3] |
| Skunk (*Mephitis mephitis*) | AB | 6 | 124 | ND | [2] |
| Wolf (*Canis lupus*) | NT | 13 | 8 | ND | [1] |
|  | YT, NT | 47 | 153 | ND | [8] |
|  | NT | 52 | 27 | T2 (6), T6 (5) | [4] |
|  | AB | 33 | 3 | ND | [1] |
|  | QC | 50 | 2 | ND | [1] |
|  | NL | 4 | 48 | ND | [1] |
|  | YT, BC, NU | 43 | 28 | T2 | [2] |
|  | AK | 37 | 148 | ND | [9] |
|  | AK | 33 | 154 | ND | [3] |

ND= Not done, NM= number not mentioned, AB=Alberta, AK=Alaska, BC=British Columbia, MB=Manitoba, NS=Nova Scotia, NT= Northwest Territories, NU=Nunavut, QC=Quebec, SK= Saskatchewan, YT=Yukon.

Table S2 Prevalence and larval burden (larvae per gram) of *Trichinella* spp. in wolverine from different geographical locations.

| **Geographical Location** | **No. tested** | **No. (%) positive** | **Method (specimen)-muscle weight** | **Mean larval burden (Range), Median^$^** | **Genotypes** | **Reference** |
| --- | --- | --- | --- | --- | --- | --- |
| North America |  |  |  |  |  |  |
| YT | 338 | 262(78) | MsDig (Tng, Dia)-10 | 22.6 (0.1-295), 8^$^ | T2, T6, T1, T13 | Present study |
| BC | 4** | 2 | MsDig/Comp (Tng, Dia, MsOther)-NM | NM | ND | [10] |
| MB | 1** | 1 | NM | NM | ND | [11] |
| NT | 38 | 0 (0) | Comp | NA | NA | [12] |
| NT | 49 | 12 (24.5) | MsDig (Tng) | 129.9/10=13 | ND | [13] |
| NU | 41 | 36 (87.8) | MsDig (Dia)-5 | 8.6 (0.2-51.8) | T2, T6 | [14] |
| NT*, BC, YT | 111 | 85 (76.6) | MsDig (Tng, Dia, MsOther)-10 | 20.8 (0.025-522), 3.7^$^ | *T2, T6 | [2] |
| AK | 38 | 19 (50) | MsDig (NM)- NM | 3.5 (0.2-18) | ND | [3] |
| Iowa | 1** | 1 | MsDig -BaTech (Tng, Dia, MsOther) | NM (10) | ND | [15] |
| Europe |  |  |  |  |  |  |
| Sweden | 27 | 0(0) | MsDig /Comp (Dia, MsOther)-NM | NA | ND | [16] |
| Asia |  |  |  |  |  |  |
| Kamchatka (Eastern  Russia)* |  | (30) |  |  |  | [17] |
| Chukotka Peninsula  Yakutia | 1** | 1 | MsDig (NM)-NM | NM | T2 | [18] |
| Yakutia | 1** | 1 | NM | NM | T1 | [19] |

AK=Alaska, BC=British Columbia, MB= Manitoba, NT= Northwest Territories, NU= Nunavut, YT=Yukon,

BaTech= Baermann technique, Comp=compressorium, MsDig=Muscle digestion, Dia=Diaphragm, MsOther= muscle other than diaphragm and tongue, Tng=Tongue,

T1= *T. spiralis*, T2= *T. nativa*, T6= *Trichinella* T6, T13= *T. chanchalensis*

NA- Not applicable, NM=Not mentioned, ND= Not done,

* All information was not extracted as published literature was not in English. ** Percentage positivity was not calculated when samples tested were less than 10.

**References:**

1. Smith HJ, Snowdon KE. Sylvatic trichinosis in Canada. Can J Vet Res. 1988;52:488–9.

2. Gajadhar AA, Forbes LB. A 10-year wildlife survey of 15 species of Canadian carnivores identifies new hosts or geographic locations for *Trichinella* genotypes T2, T4, T5, and T6. Vet Parasitol. 2010;168:78–83.

3. Rausch R., Babero BB., Rausch RV., Schiller EL. Studies on the helminth fauna of Alaska. XXVII. The occurrence of larvae of *Trichinella* *spiralis* in Alaskan mammals. J Parasitol. 1956;42:259–71.

4. Larter NC, Forbes LB, Elkin BT, Allaire DG. Prevalence of *Trichinella* spp. in black bears, grizzly bears, and wolves in the dehcho region, northwest territories, canada, including the first report of t. nativa in a grizzly bear from Canada. J. Wildl. Dis. 2011. p. 745–9.

5. Frechette, J.L,; Rau M. Helminths of the Black Bear in Quebec. J Wildl Dis. 1977;13:432–4.

6. Chomel BB, Kasten RW, Chappuis G, Soulier M, Kikuchi Y. Serological survey of selected canine viral pathogens and zoonoses in grizzly bears (*Ursus arctos horribilis*) and black bears (*Ursus americanus*) from Alaska. Rev Sci Tech. 1998;17:756–66.

7. Choquette LP, Gibson GG, Pearson AM. Helminths of the grizzly bear, *Ursus arctos* L., in northern Canada. Can J Zool. 1969;47:167–70.

8. Choquette LP, Gibson GG, Kuyt E, Pearson AM. Helminths of wolves, *Canis lupus* L., in the Yukon and Northwest Territories. Can J Zool. 1973;51:1087–91.

9. Zarnke RL, Worley DE, Ver Hoef JM, McNay ME. *Trichinella* sp. in wolves from interior Alaska. J Wildl Dis. 1999;35:94–7.

10. Schmitt N, Saville JM, Friis L, Stovell PL. Trichinosis in British Columbia wildlife. Can J Public Heal. 1976;67:21–4.

11. Chadee KC, Dick TA. Biological characteristics and host influence on a geographical isolate of *Trichinella* (wolverine: 55°00’N, 100°00’W 1979). J Parasitol. 1982;68:451–6.

12. Addison EM, Boles B. Helminth parasites of wolverine, *Gulo gulo*, from the district of Mackenzie, Northwest Territories. Can J Zool. 1978;56:2241–2.

13. Larter N, Elkin B, Allaire D. Prevalence of *Trichinella* spp . in Wildlife of the Dehcho. Goverment Northwest Territ. 2016.

14. Reichard M V., Torretti L, Snider TA, Garvon JM, Marucci G, Pozio E. *Trichinella* T6 and *Trichinella nativa* in Wolverines (*Gulo gulo*) from Nunavut, Canada. Parasitol Res. 2008;103:657–61.

15. Zimmermann WJ, Hubbard ED, Schwarte LH, Biester HE. *Trichinella spiralis* in Iowa Wildlife during the Years 1953 to 1961. J Parasitol. 1962;48:429.

16. Mörner T, Eriksson H, Bröjer C, Nilsson K, Uhlhorn H, Ågren E, et al. Diseases and mortality in free-ranging brown bear (*Ursus arctos*), gray wolf (*Canis lupus*), and Wolverine (*Gulo gulo*) in Sweden. J Wildl Dis. 2005;41:298–303.

17. Britov A. Trichinellosis in Kamchatka. Wiadomości Parazytol. 1997;43:287–8.

18. Goździk K, Odoevskaya IM, Movsesyan SO, Cabaj W. Molecular identification of *Trichinella* isolates from wildlife animals of the Russian Arctic territories. Helminthol. 2017;

19. Odoevskaya IM, Spiridonov SE. Molecular taxonomic study of *Trichinella* spp. from mammals of Russian Arctic and subarctic areas. Czech Polar Reports. 2014;4:40–6.
